# Insight into the new infection pathway resulting from above-ground pathogen infection of grapevine crown gall

**DOI:** 10.3389/fpls.2024.1420401

**Published:** 2024-09-30

**Authors:** Akira Kawaguchi

**Affiliations:** Western Region Agricultural Research Center (WARC) (Kinki, Chugoku and Shikoku Regions), National Agriculture and Food Research Organization (NARO), Fukuyama, Hiroshima, Japan

**Keywords:** *Allorhizobium vitis*, spatiotemporal distribution, grapevine crown gall, secondary infection, binary power law

## Abstract

Grapevine crown gall (GCG), a soil-borne plant disease caused by tumorigenic *Allorhizobium vitis* (TAV) (=tumorigenic *Rhizobium vitis*) strains, poses a significant threat to grapevines worldwide. Recently, outbreaks of GCG have been reported in several vineyards, necessitating investigation into potential alternative infection pathways beyond soil transmission. The spatiotemporal distribution of GCG in vineyards from 2020 to 2022 was analyzed using the binary power law (BPL) model, with variations in quadrat shapes. Both total and newly observed diseased plants exhibited an aggregated distribution, indicating that new infections clustered around existing diseased plants, with secondary infections appearing as independent cluster points. This study provides evidence that infected pruning tools can transmit the pathogen to healthy grapevines and that TAV inoculation through spraying contributes more to GCG incidence than planting in infected soil alone. This represents the first documented case of secondary above-ground TAV infection contributing to GCG in commercial vineyards.

## Introduction

Grapevine (*Vitis vinifera* L.) crown gall (GCG) is primarily caused by tumorigenic *Allorhizobium vitis* (TAV) [syn. tumorigenic *Rhizobium vitis*, tumorigenic *Agrobacterium vitis* (Ti), and *A. tumefaciens* biovar 3] (Mousavi et al., 2015). TAV infects grapevines through various wounds, including freezing injuries, cutting damage, and grafting (Burr et al., 1998; Burr and Otten, 1999; Kawaguchi, 2022b; Kawaguchi et al., 2017, 2021, 2023b; Gan et al., 2019). GCG presents a global challenge, with galls typically emerging on both mature and young grapevine trunks and cordons, including nursery stocks (Burr et al., 1998; Kawaguchi, 2009; Kawaguchi et al., 2017, 2021, 2023b; Gan et al., 2019). Infected grapevines often exhibit inferior growth, and in severe cases, the galls lead to grapevine death (Kawaguchi, 2022a).

The primary objective of this study is to harness the antagonistic potential of these strains to develop a new biopesticide, urgently needed as GCG frequently occurs in Japan (Kawaguchi et al., 2023a). Particularly, in recent years, severe damage from GCG has been reported in several vineyards in northern regions of Japan (Kawaguchi et al., 2023a). Interestingly, numerous observations suggest the occurrence of secondary TAV infections in commercial vineyards. However, there have been no reports on alternative infection pathways besides soil transmission and planting infected nursery stocks thus far (Burr et al., 1998). Therefore, the objective of this study is to uncover other potential infection pathways contributing to GCG outbreaks in commercial vineyards. This article presents the findings of investigations into GCG occurrence in infected vineyards, spatiotemporal distribution of GCG, and the efficiency of TAV infection through above-ground processes.

## Materials and methods

### Field survey

**Figure 1:**
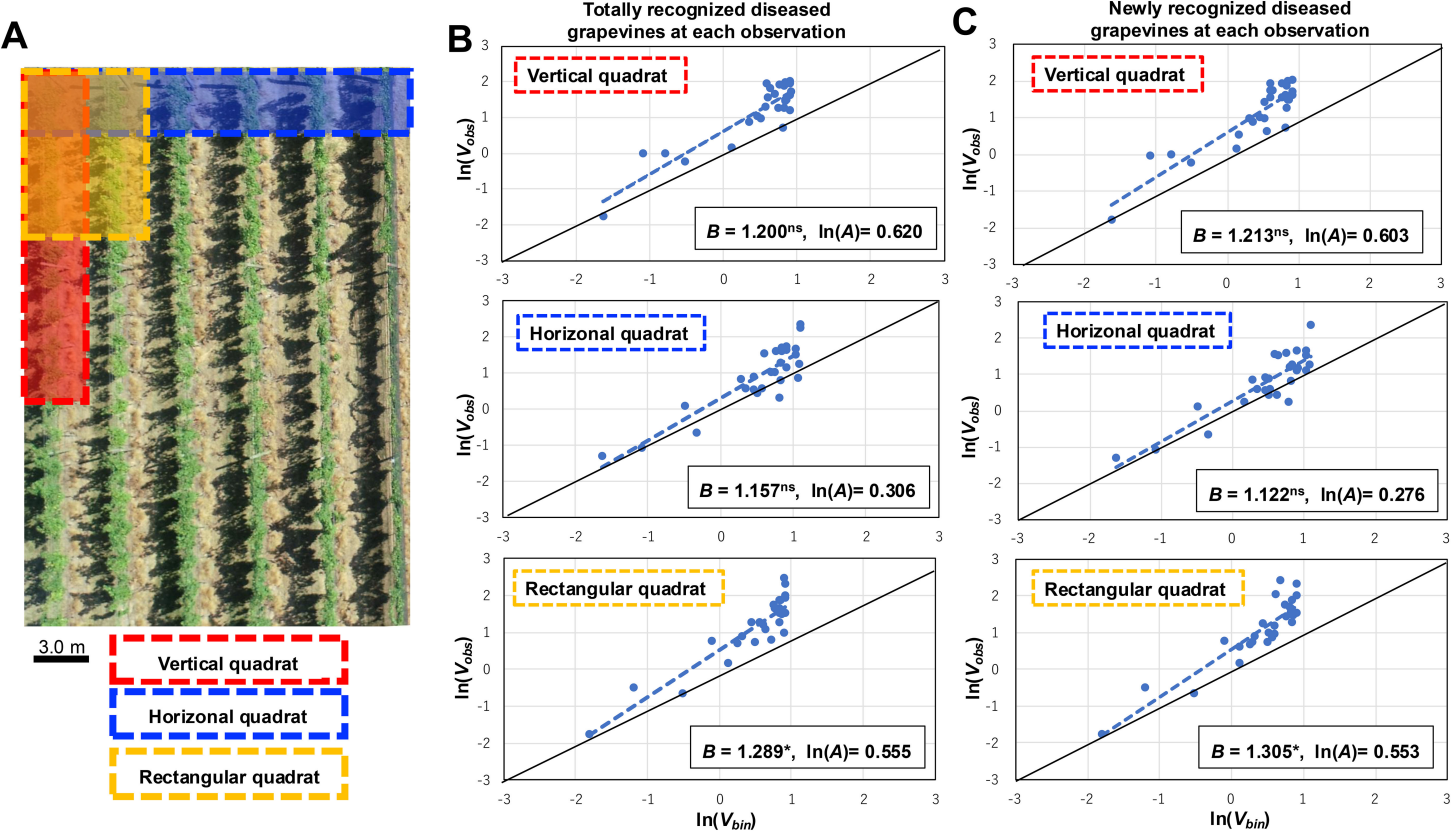
Spatiotemporal distribution analysis of grapevines naturally infected with grapevine crown gall (GCG) in commercial vineyards includes the following components: **(A)** Placement of three distinct quadrat patterns within each vineyard. An aerial image illustrates a section of the vineyard. These patterns consist of a vertical quadrat comprising a row of 10 plants (highlighted in red), a horizontal quadrat spanning 10 plants across each row (highlighted in yellow), and a rectangular quadrat comprising two adjacent rows of 5 plants each, totaling 10 plants per quadrat (highlighted in blue); **(B)** Regression analysis conducted for totally recognized diseased plants during each observation using the binary power law (BPL); **(C)** Regression analysis performed for newly recognized diseased plants during each observation using the BPL. The dashed line represents the BPL regression line, while the solid line depicts ln(*V_obs_*) = ln(*V_bin_*) (*B =*1, ln(*A*) = 0), indicating a random distribution. The B values derived from the BPL regressions were significantly above 1.0, denoted by * for *P* < 0.05, and ^ns^ for *P*> 0.05.

The spatial spread patterns of GCG within 20 vineyards over the past three years since 2020 were investigated, with detailed information provided for each vineyard in **Supplementary Tables S1**, **S2**. Although vineyard sizes varied, investigation plots were similar, ranging from a maximum of 120 × 30 m to a minimum of 60 × 15 m. Rows within each vineyard were approximately 3 m apart. GCG incidence was assessed and mapped in contiguous quadrats, covering 4 to 6 rows of 200 to 360 grapevines. Three different quadrat patterns were employed in each vineyard: first, a vertical quadrat consisting of a single row of 10 plants; second, a horizontal quadrat comprising 10 plants crossing multiple rows (not following a specific row pattern); and third, a rectangular quadrat consisting of two or three adjoining rows of 5 plants each, totaling 10 to 15 plants per quadrat (**Figure 1A**). The number of plants exhibiting GCG symptoms was recorded in each vineyard. To confirm the presence of TAV, symptomatic plant tissues (galls) were randomly sampled from the vineyards. Colonies generated on AV selective medium, namely Roy and Sasser (RS) medium (Roy and Sasser, 1983; **Supplementary Table S3**), were subjected to confirmation through multiplex PCR using two TAV-specific primer sets: Ab3-F3/Ab3-R4 and VCF3/VCR3, as previously reported (Kawaguchi et al., 2005a; **Supplementary Table S4**).

### Mathematical analysis

The binary power law (BPL) serves as a model for characterizing the spatial heterogeneity of plant disease incidence (Hughes and Madden, 1992, 1993; Madden et al., 2018). When utilizing *x_i_*, the observed variance (*V_obs_*) and the variance of a binominal distribution (*V_bin_*) are defined as follows:


(1) $$p=\sum_{i=1}^{n}x_{i}/N$$

(2) $$V_{obs}=\sum_{i=1}^{n}{\left(x_{i}-np\right)}^{2}/\left(n-1\right)$$

(3) $$V_{bin}=np(1–p)$$

where $\sum_{i=1}^{n}x_{i}$ is the number of diseased plants, *n* is the number of quadrats, *x_i_* is the number of diseased plants in the *i* th quadrat (*i =*1…*n*), *p* is a parameter representing the probability of an individual being diseased in field and *N* is a total number of investigated plants. Furthermore, a relationship between *V_obs_* and *V_bin_* was defined as follows (Hughes and Madden, 1992; Madden et al., 2018):


(4) $$V_{obs}=A\;V_{bin}^{B}$$

(5) $$\text{ln}\;\left(V_{obs}\right)=\text{ln}\;\left(A\right)+B\;\text{ln}\;\left(V_{bin}\right)$$

Where A and B are parameters, with B representing the exponent of the BPL, serving as the slope of the straight-line relationship between the logarithm of *V_obs_* and the logarithm of *V_bin_*. When *A* = *B* = 1, the BPL reduces to *V_obs_* = *V_bin_*, indicating that the observed variance equals the variance that the data would have if xi followed a binomial distribution (Hughes and Madden, 1992; Madden et al., 2018). When *A* > 0 and *B* = 1, overdispersion occurs, independent of the level of disease incidence (*p*), suggesting that the degree of heterogeneity remains constant for all incidence values (Hughes and Madden, 1992; Madden et al., 2018). If *B* does not equal 1, overdispersion (heterogeneity) systematically varies with the level of incidence (Hughes and Madden, 1992; Madden et al., 2018). Typically, *B* > 1 indicates that *V_obs_* increases faster than *V_bin_*, demonstrating an aggregation distribution (Hughes and Madden, 1992; Madden et al., 2018). In this study, when *B* is significantly greater than 1.0 (*p* < 0.05), diseased plants demonstrate aggregation distribution.

### Population measurement of TAV in grape shoots

To monitor the population dynamics of TAV following different inoculation methods, inoculation and re-isolation were performed. Grapevine seedlings (2 years old, cv. Muscat of Alexandria) were grown from seeds, which were provided some commercial vineyards without GCG incidence in Okayama prefecture, Japan. Seedling grown by seed were used because potential natural contamination of TAV in commercial nursery stocks should be avoided in this study (Burr et al., 1998). A cell suspension of TAV strain VAT20-1sc at a concentration of 10^5^ cells/ml was prepared. VAT20-1sc is a streptomycin (St)- and copper sulfate (CuSO_4_)-resistant mutant of VAT20-1, which was isolated from a galled grapevine in 2020 in Hokkaido, Japan (Kawaguchi et al., 2021), obtained by culturing on PDA medium (Difco, Detroit, MI, USA) containing 500 μg/ml St and 250 μg/ml CuSO_4_ (Kawaguchi, 2015).

For contaminated scissors inoculation, scissors were briefly soaked in the TAV cell suspension for 1 second, and then used to cut stems of 70 plants for inoculation. For spray inoculation, which mimicked TAV infection from diseased plants by water splashing in vineyards, stems of plants were cut using non-infected scissors, and the plants were then sprayed with the TAV cell suspension (5 ml per plant). Inoculated plants were cultivated in a greenhouse at 25°C.

To confirm TAV infection at 1, 3, 5, 9, 21, and 31 days after inoculation (dai), 10 plants (i.e., *n* = 10) were randomly selected from the inoculated plants at each sampling day. Stem samples were collected, including the inoculation point of the plants (0.1 g fresh weight per plant, 1 sample per plant), and TAV populations were assessed using the serial dilution plate method on RS medium containing St and CuSO_4_ following the procedure previously reported (Kawaguchi et al., 2023b). As negative controls, five healthy grapevine seedlings (i.e., *n* = 5) were prepared, and TAV strain isolation was conducted.

### Disease incidence by three different inoculation methods

To prepare infected soil, strain VAT20-1 was inoculated into the soil by pouring cell suspensions at a concentration of 10^8^ cells/ml onto the soil (500 ml/kg soil), resulting in a final concentration of approximately 5 × 10^7^ cells/g soil (Kawaguchi and Inoue, 2012). The roots of grapevine seedlings were pruned by half and then planted in pots (15 seedlings per pot) containing the infected soil.

For spray or contaminated scissors inoculation, two concentrations (10^5^ and 10^7^ cells/mL) of TAV strain VAT20-1 cell suspension were prepared. In contaminated scissors inoculation, scissors were briefly immersed in the TAV cell suspension for 1 second, and then used to cut stems of 15 plants (i.e., *n* = 15) for inoculation. In spray inoculation, which simulated natural conditions, stems of 15 plants were cut using non-infected scissors, and then the plants were sprayed with the TAV cell suspension (5 ml per plant). Inoculated plants were cultivated in a greenhouse at 25°C, and gall formation on the roots and stem wounds of seedlings was assessed after 3 to 4 months. The inoculation experiment was independently repeated 5 times. TAV inoculated into the plants was re-isolated using RS medium from formed galls in each experiment, and some re-isolated colonies on RS medium were confirmed as VAT20-1 by rep-PCR DNA fingerprinting, using the (GTG)_5_ primer set following the procedure previously reported (Wong et al., 2021).

### Data analysis

All statistical analyses were performed using the RStudio user interface (version 1.2.5001) for R software (version 4.3.1, R Foundation for Statistical Computing, http://www.r-project.org/)“.

## Results

### Spatiotemporal distribution of grapevines with GCG symptom in commercial vineyards

The spatiotemporal distribution of GCG in vineyards from 2020 to 2022 was analyzed using the BPL with varying quadrat shapes. The results of the BPL applied to the combined data for the total number of recognized diseased grapevines in each vineyard revealed the observed variance (*V_obs_*) and the variance of a binomial distribution (*V_bin_*) (**Figure 1B**, **Table 1**). Based on equation (5), estimates of *B* and ln(*A*) were obtained for three types of quadrat shapes, with the estimated B in the rectangular quadrat being 1.289 (95% confidence interval (CI): 1.046 to 1.533), significantly greater than 1.0 (*p* < 0.05) (**Figure 1B**, **Table 1**). Additionally, the *V_obs_* of newly recognized diseased plants at each observation increased with *V_bin_* (**Figure 1C**). Similarly, for the totally recognized diseased plants, the estimated *B* in the rectangular quadrat was 1.305 (95% CI: 1.067 to 1.543), also significantly greater than 1.0 (*p* < 0.05) (**Figure 1C**, **Table 1**). These results indicate that both totally and newly observed diseased plants exhibited an aggregated distribution, suggesting that new diseased plants caused by secondary infections clustered around previous observed diseased plants, while secondary infections occurred as independent cluster points.

**Table 1:** Slope and intercept parameter estimates of binary power law (BPL) for incidence of grapevines with crown gall symptoms from data sets collected from commercial vineyards.

Data set	Quadrat shape	*B*	se	95% confidencial interval (CI)		ln(*A*)	se	95% confidencial interval (CI)		*R* ^2^	*P* value of *R* ^2^
				Lower	Upper			Lower	Upper		
Totally recognized diseased plants at each observation	Vertical quadrat	1.200	0.111	0.972	1.427	0.620	0.089	0.437	0.803	0.819	4.0×10^-11^
	Horizontal quadrat	1.157	0.124	0.902	1.413	0.306	0.306	0.089	0.523	0.770	9.0×10^-10^
	Rectangular quadrat	1.289	0.118	1.046	1.533	0.555	0.094	0.362	0.750	0.820	3.5×10^-11^
Newly recognized diseased plants at each observation	Vertical quadrat	1.213	0.117	0.973	1.452	0.603	0.088	0.423	0.784	0.806	9.3×10^-11^
	Horizontal quadrat	1.122	0.172	0.875	1.370	0.276	0.096	0.079	0.474	0.770	8.9×10^-10^
	Rectangular quadrat	1.305	0.116	1.067	1.543	0.553	0.087	0.375	0.731	0.829	8.9×10^-10^

### Population of TAV in grapevine shoots in vineyards

**Figure 2:**
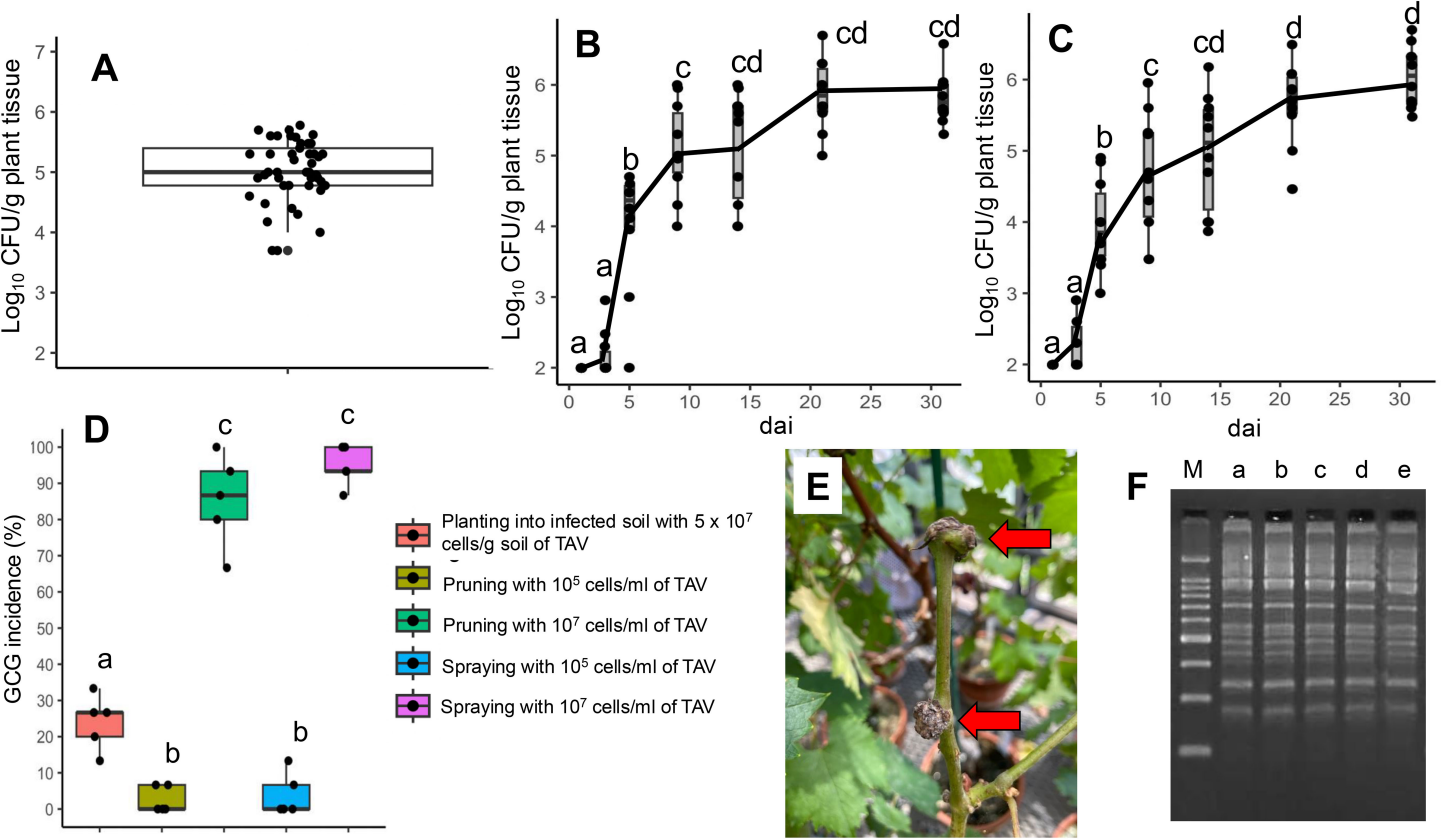
Populations of tumorigenic *Allorhizobium vitis* (TAV) and grapevine crown gall (GCG) incidence: **(A)** TAV population in grapevine shoots within vineyards; **(B)** Dynamics of TAV populations in grapevine shoots post-inoculation via pruning with contaminated scissors using a TAV cell suspension (10^5^ cells/ml); **(C)** Dynamics of TAV populations in grapevine shoots post-inoculation via spraying with a TAV cell suspension (10^5^ cells/ml); **(D)** Evaluation of GCG incidence resulting from various inoculation methods and concentrations of cell suspensions; **(E)** Observation of GCG symptoms following spray inoculation with a TAV cell suspension (10^7^ cells/ml), with red arrows indicating gall formations; **(F)** Results of agarose gel electrophoresis of rep-PCR DNA fingerprinting using the (GTG)_5_ primer set, with ‘M’ representing the DNA marker, ‘a’ indicating VAT20-1, ‘b’ and ‘c’ representing re-isolated strains from galls resulting from spraying with 10^5^ cells/ml of VAT20-1, ‘d’ and ‘e’ representing re-isolated strains from galls resulting from pruning with 10^5^ cells/ml of VAT20-1, and demonstrating consistent band patterns across lanes from ‘a’ to ‘e’. In **(B–D)**, the median is denoted by the center bar of the box plot, with the lower and upper horizontal bars indicating the 25th and 75th percentiles respectively. The whiskers represent the 95% range. In the population dynamics of TAV, boxes labeled with different letters denote a significant difference from other boxes (*p* ≤ 0.05, Tukey’s HSD test). In GCG incidence (%), boxes labeled with different letters indicate a significant difference from other boxes (*p* ≤ 0.05, Ryan’s test).

The CFU (colony-forming units) of TAV were detected in grapevine shoot samples obtained from grapevines displaying GCG symptoms in three different commercial vineyards. An average of 5.02 ± 0.51 log_10_ CFU/g plant tissue (mean ± standard deviation of the mean) (95% CI; 4.88 to 5.17) was observed (**Figure 2A**), indicating that approximately 10^5^ cells/g plant tissue of TAV could inhabit shoots in naturally infected grapevines.

### Population dynamics of TAV in grapevine shoots after inoculation

Following inoculation by spraying or pruning using contaminated scissors with a cell suspension of TAV (10^5^ cells/ml), TAV exhibited rapid growth in grapevine shoots by 10 days after inoculation (dai), with similar dynamics observed among different inoculation methods (**Figures 2B, C**). At 31 dai, 5.82 ± 0.36 log_10_ CFU/g plant tissue (95% CI; 5.60 to 6.10) was detected with pruning inoculation, while 6.04 ± 0.43 log_10_ CFU/g plant tissue (95% CI; 5.76 to 6.31) was detected with spray inoculation (**Figures 2B, C**). These findings suggest that even relatively low numbers of TAV cells, such as 10^5^ cells/ml through above-ground inoculation methods, could infect and proliferate within grapevines.

### Assessment of GCG incidence resulting from different inoculation methods and concentrations of cell suspension

GCG incidence was evaluated by comparing incidences of diseased (galled) plants following inoculation by different methods, including planting into infected soil with 5 ×10^7^ cells/g soil, pruning with contaminated scissors soaked in 10^5^ and 10^7^ cells/ml TAV cell suspensions, and spraying with 10^5^ and 10^7^ cells/ml TAV cell suspension. When pruning and spraying with 10^5^ cells/ml TAV cell suspension were employed, GCG incidences were 2.7% and 4.0%, respectively (**Figure 2D**). However, using a high concentration of cell suspension (10^7^ cells/ml), the number of diseased plants significantly increased, with GCG incidences following pruning and spraying reaching 85.3% and 94.7%, respectively (**Figures 2D, E**). Some re-isolated strains were confirmed as strain VAT20-1 (inocula) by rep-PCR DNA fingerprinting (**Figure 2F**). In contrast, planting into infected soil resulted in a GCG incidence of 24.0% and significantly lower than spray and pruning inoculation (10^7^ cells/ml) (**Figure 2D**). These results suggest that the likelihood of GCG incidence through above-ground inoculation methods, such as pruning and spraying, was higher than that through planting into infected soil.

## Discussion

In this study, spatiotemporal distribution analysis of GCG using BPL was conducted, revealing an aggregated distribution of both totally and newly observed diseased grapevines. If GCG occurred solely through soil infection or infected nursery stocks without any other transmission of TAV, newly observed diseased plants would not appear around previously affected plants. Thus, these findings suggest that new infections formed cluster points around diseased plants, while secondary infections occurred as independent cluster points. This marks the first report indicating an aggregated distribution of plants with GCG symptoms in commercial vineyards, indicating at potential alternative pathogen transmission pathways.

Certain soil-borne bacterial diseases, such as tomato bacterial canker (TBC) and bacterial black node of barley and wheat, exhibit multiple routes of infection. Pathogens can transfer from diseased plants to healthy ones through water splash or agricultural practices like pruning (Kawaguchi et al., 2010, 2013, 2018; Peritore-Galve et al., 2021). Notably, in the case of TBC, disbudding and defoliation significantly contribute to the secondary spread of the disease in commercial greenhouses (Kawaguchi et al., 2010, 2013). If disbudding and defoliation contribute to transmit pathogen to healthy plants, disease plants should aggregate in vertical quadrats (Kawaguchi et al., 2010, 2013). If pathogen could widely spread by strong wind or rain, it likely seems diseased plants should aggregate in horizonal quadrats and/or other shape quadrats. In this study, However, diseased plants aggregated and formed clusters within rectangular-shaped quadrats, which were relatively smaller than other quadrats. This suggests that the transmission range of TAV may be limited, with the pathogen spreading primarily to neighboring diseased plants (**Figure 1**). Consequently, activities like splashing water and pruning with contaminated scissors could facilitate secondary above-ground spread, akin to TBC.

Pruning is one of the fundamental agricultural practices in vineyards. Shoots from diseased plants contained approximately 10^5^ cells/g of plant tissue (**Figure 2A**). When a grower prunes a healthy grapevine after having pruned a diseased one, 10^5^ cells/ml of TAV, which are attached to the scissors, could infect and incubate inside the shoot (**Figure 2B**). However, the incidence of GCG was only 2.7% through pruning, significantly lower than those inoculated by other methods with 10^7^ cells/ml of TAV (**Figure 2D**). Similarly, spraying with 10^5^ cells/ml of TAV, which mimicked TAV transmission from diseased grapevines by water splashing in vineyards, incubated at the wounds of grapevines resulted in only a 4.0% incidence of GCG, similar to pruning with the same TAV concentration (**Figures 2C, D**). TAV, present at around 10^4^ cells/g of plant tissue, is found on/in the skins of diseased grapevines (Kawaguchi et al., 2023b). Based on these results and a previous report, even if splashing water occurs due to rain or watering, facilitating the transmission of TAV from diseased grapevines to neighboring healthy ones, the spread of TAV may not directly contribute to GCG incidence in vineyards. However, TAV could proliferate at grapevine wounds after being spread and attached. This secondary spread through splashing water and pruning shoots could potentially lead to TAV infection, with GCG manifesting under favorable environmental conditions in the future.

On the other hand, gall tissues of grapevines, occurring through natural infection in vineyards, contain approximately 10^7^ cells/g of TAV (Kawaguchi et al., 2023b). The results of spraying (mimicked water splashing in vineyards) and pruning (mimicked grower’s agricultural practices by pruning after handling gall tissues) with 10^7^ cells/ml of TAV indicate that growers could inadvertently spread TAV by pruning after handling gall tissues, and that water splashing could facilitate the spread of TAV from gall tissues of grapevine to healthy tissues of the same grapevine and/or neighboring grapevines (**Figure 2D**). Some growers attempt to scrape off galls with a knife in commercial vineyards, but it is imperative for them to disinfect the contaminated knife to prevent further spread.

GCG is recognized as a soil-borne disease (Burr et al., 1998). However, in this study, the incidence of GCG when planting into soil contaminated with TAV (5 × 10^7^ cells/g soil) was significantly lower than when TAV was applied through spraying and pruning (cell concentration of 10^7^ cells/ml) (**Figure 2D**). This suggests that secondary infection through splashing water and/or pruning with contaminated knives after scraping galls could lead to severe GCG incidence, such as over 50% GCG incidence in vineyards. These results strongly support the notion that the initial diseased plant, caused by soil infection and/or the planting of already infected nursery stocks without visible GCG symptoms, acts as the primary inoculum. Furthermore, inocula from diseased plants are also likely to cause secondary infection with TAV through splashing water and/or pruning with severely contaminated knives in neighboring healthy plants in commercial vineyards. Specifically, splashing water could transport TAV from galls to various wounds, including freezing injuries, cuts, and/or mechanical damages on grapevines.

In any case, the roles of primary and secondary inocula in the disease cycle in fields remain unclear. Thus, clarifying these roles is crucial as it could enable farmers to control the disease more efficiently. To assess the significance of primary and secondary inocula, the author investigated the spatiotemporal distribution of grapevines naturally infected with TAV in vineyards following the initial detection of the disease. This investigation involved comparisons using an epidemiological model BPL and various quadrat shapes. This study revealed that populations of the TAV pathogen formed new independent clusters during outbreaks. To directly address this hypothesis, labeling TAV strains by inserting antibiotic or fluorescence genes and investigating GCG outbreaks in greater detail could be beneficial. However, conducting such experiments in open-air commercial vineyards presents significant challenges. Thus, a statistical analysis of spatiotemporal distribution becomes essential for planning efficient disease management strategies. Based on these findings, growers may need to consider spraying bactericides in vineyards to control secondary infections and prevent GCG outbreaks.

The absence of an effective and practical GCG management method poses a significant issue. Previous studies have highlighted that nonpathogenic and antagonistic *A. vitis* strains, such as ARK-1 and VAR03-1, inhibited gall formation not only in grapevines but also in various plant species (Kawaguchi, 2009, 2011, 2013, 2014, 2015; Kawaguchi et al., 2005b, 2007, 2008, 2012, 2015, 2017, 2019, 2023a; Kawaguchi and Inoue, 2012; Kawaguchi and Noutoshi, 2022a, 2022b; Kawaguchi, 2022b; Ishii et al., 2024; Noutoshi et al., 2020; Saito et al., 2018; Wong et al., 2021). While there are currently no registered bactericides available for growers to apply in their own vineyards, a new biopesticide made from strain ARK-1 is currently under development (Kawaguchi, 2022b). Spraying this new biopesticide onto grapevine cordons above ground could effectively prevent GCG incidence resulting from secondary infections.

In conclusion, TAV exhibits a secondary infection pathway above ground and seems to initiate new infections from the primary inoculum at the beginning of an outbreak. Subsequently, each new infection caused by secondary infection leads to another round of new infections, ultimately expanding the area of diseased grapevines in the disease cycle within commercial vineyards. Currently, there are no reports available on the pattern of TAV infection spread in vineyards. This study offers evidence to infer both the source and mode of spread of TAV, which causes GCG.

## Data Availability

The datasets presented in this study can be found in online repositories. The names of the repository/repositories and accession number(s) can be found in the article/**Supplementary Material**.
